# Interior and Edge Magnetization in Thin Exfoliated CrGeTe_3_ Films

**DOI:** 10.1021/acs.nanolett.1c04665

**Published:** 2022-03-10

**Authors:** Avia Noah, Hen Alpern, Sourabh Singh, Alon Gutfreund, Gilad Zisman, Tomer D. Feld, Atzmon Vakahi, Sergei Remennik, Yossi Paltiel, Martin Emile Huber, Victor Barrena, Hermann Suderow, Hadar Steinberg, Oded Millo, Yonathan Anahory

**Affiliations:** † Racah Institute of Physics, The Hebrew University, Jerusalem 91904, Israel; ‡ Department of Applied Physics, The Hebrew University of Jerusalem, Jerusalem 91904, Israel; § Center for Nanoscience and Nanotechnology, Hebrew University of Jerusalem, Jerusalem 91904, Israel; ∥ Departments of Physics and Electrical Engineering, University of Colorado Denver, Denver, Colorado 80217, United States; ⊥ Laboratorio de Bajas Temperaturas, Unidad Asociada UAM/CSIC, Departamento de Física de la Materia Condensada, Instituto Nicolás Cabrera and Condensed Matter Physics Center (IFIMAC), Universidad Autónoma de Madrid, E-28049 Madrid, Spain

**Keywords:** 2D magnetism, edge magnetism, superconducting/ferromagnetic interface, scanning SQUID-on-tip microscopy, CrGeTe_3_

## Abstract

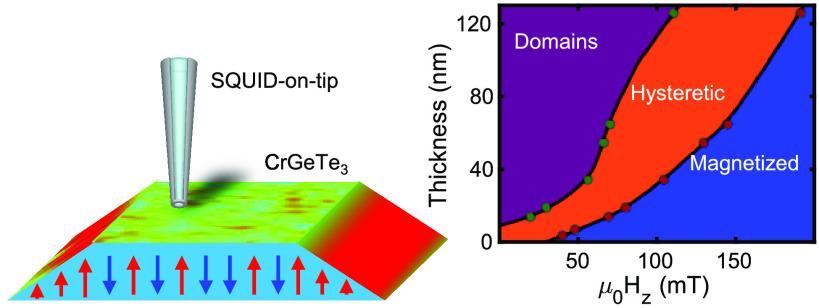

CrGeTe_3_ (CGT) is a semiconducting vdW ferromagnet shown to possess magnetism down to a two-layer thick sample. Although CGT is one of the leading candidates for spintronics devices, a comprehensive analysis of CGT thickness dependent magnetization is currently lacking. In this work, we employ scanning SQUID-on-tip (SOT) microscopy to resolve the magnetic properties of exfoliated CGT flakes at 4.2 K. Combining transport measurements of CGT/NbSe_2_ samples with SOT images, we present the magnetic texture and hysteretic magnetism of CGT, thereby matching the global behavior of CGT to the domain structure extracted from local SOT magnetic imaging. Using this method, we provide a thickness dependent magnetization state diagram of bare CGT films. No zero-field magnetic memory was found for films thicker than 10 nm, and hard ferromagnetism was found below that critical thickness. Using scanning SOT microscopy, we identify a unique edge magnetism, contrasting the results attained in the CGT interior.

## Introduction

Layered van der Waals (vdW) ferromagnets have recently been the focus of intensive research due to the easily accessible broad thickness range they offer, from the bulk material all the way to atomically thin two-dimensional (2D) crystals, enabled by exfoliation. While the revolution triggered by the vdW materials is well underway,^1−4^ the emerging field of 2D vdW spintronics is still in its infancy.^5−7^ The need for compatible materials with long-range ferromagnetic order and precise analysis of such materials are at the core of this new emerging field. The evolution of the magnetic properties from bulk material to thin exfoliated layers may offer additional insight into the origin of ferromagnetism in vdW materials, where anisotropy was suggested^8^ to originate from distinct interlayer and intralayer exchange interactions. Exfoliating bulk vdW ferromagnets, either conducting such as Fe_3_GeTe_2_ (FGT)^9^ or semiconducting such as CrGeTe_3_ (CGT)^8^ and CrI_3_^10^ has revealed that ferromagnetism can survive down to the few layers regime where the Mermin–Wagner theorem asserts long-range ordering should be suppressed by thermal fluctuations in the absence of magnetic anisotropy.^11^ Such anisotropy is manifested as out-of-plane (OOP) easy axis magnetization for both FGT^12^ and CGT.^13,14^ Ferromagnetism in those materials was mostly characterized using Anomalous Hall effect (AHE) measurements (that cannot be applied to the insulating CGT)^9,15−17^ and SQUID (superconducting quantum interference device) magnetometry,^12,13,18^ which average over the whole sample, or by local probes such as Kerr rotation,^8^ low-temperature magnetic force microscopy (MFM),^12^ X-ray magnetic circular dichroism (XMCD),^18^ and NV-centers.^19^ The local probe methods are highly effective for investigating edge magnetization in vdW materials, an issue that has recently attracted considerable interest.

In our present work we utilize scanning SQUID-on-tip (SOT), with high spatial resolution^20−22^ and single-electron magnetic moment sensitivity,^23,24^ in combination with transport measurements of CGT/NbSe_2_ bilayers, to provide an accurate thickness dependence of the magnetic properties of CGT flakes. Our results show that the magnetic characteristics at the flake’s edges is different from its interior. The thickness dependence of the film’s magnetic behavior can offer a control mechanism that could be used in giant magnetoresistance-like devices.

## Results

### CGT/NbSe_2_ Bilayer

Probing the magnetic properties of ferromagnetic materials using electrical measurements such as AHE is a powerful method to study samples that are too small to be characterized by bulk magnetization techniques. However, insulating materials such as CGT are not compatible with electrical measurements. Hence, so far the magnetism of CGT was characterized only indirectly by carrying out transport measurements on a conducting layer coupled to CGT, including induced AHE in proximitized Pt,^16^ topological insulator (TI),^17^ and through magnetoresistance hysteresis in a ferromagnet/superconductor CGT/NbSe_2_ bilayer.^25^

**Figure 1:**
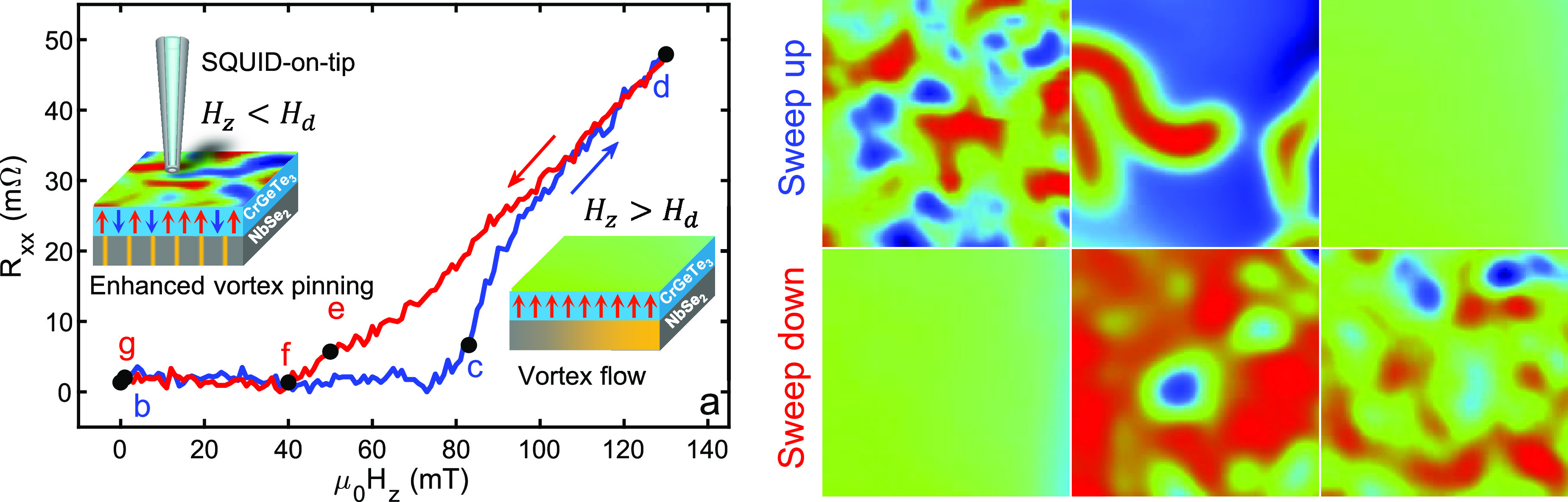
CrGeTe_3_/NbSe_2_ magnetoresistance and corresponding SOT images at 4.2K. (a) *R*_*xx*_ measurements of the NbSe_2_ layer as a function of out-of-plane (OOP) magnetic field μ_0_*H*_*z*_ while applying current *I*_*x*_ = 250 μA and sweeping the field up/down (blue/red). Left inset: Schematic illustration of the bilayer below the demagnetization field *H*_d_ = 40 mT in the enhanced vortex pinning state. Stationary vortices are depicted in orange. Right inset: Same as left but above *H*_d_ in the vortex flow (finite *R*_*xx*_) state. (b–g) Sequence of magnetic images of the OOP component of the local magnetic field *B*_*z*_(*x*,*y*) of different states of CGT at distinct values of μ_0_*H*_*z*_ acquired simultaneously with transport data in (a) (labeled black dots). (b–d) *B*_*z*_ (*x*,*y*) images acquired sweeping up the field at μ_0_*H*_*z*_ values of (b) 0, (c) 85, and (d) 130 mT. (e–g) *B*_*z*_(*x*, *y*) images acquired sweeping the field down μ_0_*H*_*z*_ = (e) 50, (f) 40, and (g) 0 mT. All images are 1 × 1 μm^2^ in size (pixel size = 20 nm); acquisition time 5 min/image. The blue to red color scale represents lower and higher magnetic fields, respectively, with a shared scale of *B*_*z*_ = (b, g) 1 mT and (c–f) 5 mT. See Supplementary Movie 1.

The CGT/NbSe_2_ sample presented in Figure 1 consists of ∼30 nm CGT flake placed on a ∼30 nm NbSe_2_ exfoliated on top of prepatterned Au contacts (see Figure S6). Figure 1a presents the longitudinal resistance (*R*_*xx*_) of the NbSe_2_ flake with constant current *I*_*x*_ = 250 μA as a function of the out-of-plane (OOP) magnetic field μ_0_*H*_*z*_ at 4.2 K. In this magnetoresistance measurement, μ_0_*H*_*z*_ was ramped up from 0 to 130 mT (blue curve) and ramped down back to 0 (red curve). A clear hysteresis is evident between μ_0_*H*_*z*_ = 40 mT and μ_0_*H*_*z*_ = 80 mT, where a switching between the dissipationless and resistive states occurs, consistent with previous measurements reported in ref (25), yet its origin was not explained.

To gain better insight into the origin of this hysteretic behavior, we conduct local magnetic field imaging *B*_*z*_(*x*,*y*) using a scanning SOT, aiming to correlate the local magnetic structure of the CGT flake and the magnetoresistance hysteresis of the bilayer. The SOT measurements were simultaneously carrying out with the transport using SOT with loop diameter ranging from 155 to 180 nm (see Methods and Supplementary Note 1). Figure 1b presents a SOT image of the CGT sample measured at a distance of ∼100 nm above the sample for μ_0_*H*_*z*_ = 0. The image resolves magnetic domain features sized lower than the tip diameter (155 nm), yielding a magnetic contrast of ∼1 mT. With increasing OOP field, domains parallel to the field grow at the expense of the antiparallel domains (Figure 1b–d and Supplementary Movie 1). Above the saturation field, μ_0_*H*_s_ ∼ 100 mT, the magnetic landscape becomes smooth with a weaker contrast. These results are consistent with the transport and global magnetization measurements of Pt/CGT(65 nm) bilayers^16^ as well as with the general behavior of bulk CGT.^13,26^ By decreasing the field, the sample’s magnetic images remain featureless down to μ_0_*H*_*z*_ = 40 mT (Figure 1d,e and 1a, right inset), where magnetic domains reappear (Figure 1f and 1a, left inset).

A clear correlation emerges between the transport measurement and the magnetic images. The magnetic texture of the CGT flake (Figure 1b) is expected to provide local pinning potentials, inhibiting flux flow, which is manifested as the zero-voltage state (Figure 1a, blue curve prior to point c). Upon saturating the CGT magnetization (Figure 1c), the pinning potential flattens, allowing flux flow that generates dissipation and hence a finite voltage. Once CGT is fully magnetized (Figure 1a, right inset), the pinning potential is sufficiently uniform to yield uninhibited flux flow manifested in a linear magnetoresistance.^27^ When reducing the field back from the saturation field, the linear magnetoresistance persists (Figure 1a, red curve), in agreement with the featureless images (Figure 1d,e). An abrupt formation of magnetic domains takes place at a demagnetization field, μ_0_*H*_d_ = 40 mT. Importantly, CGT’s demagnetization (Figure 1f) occurs simultaneously with the switching of the transport measurements back to the dissipationless state where vortices are pinned by the magnetic structure (Figure 1, left inset).

Our SOT images thus provide a clear evidence for the magnetic texture of CGT causing the hysteretic magnetoresistance observed on the CGT/NbSe_2_ bilayer (Figure 1a). Furthermore, due to the exact correlation between the transport measurements and the magnetic imaging, we demonstrate how the magnetoresistance of the CGT/NbSe_2_ bilayer could be used to globally probe the magnetic properties of the CGT flake.

**Figure 2:**
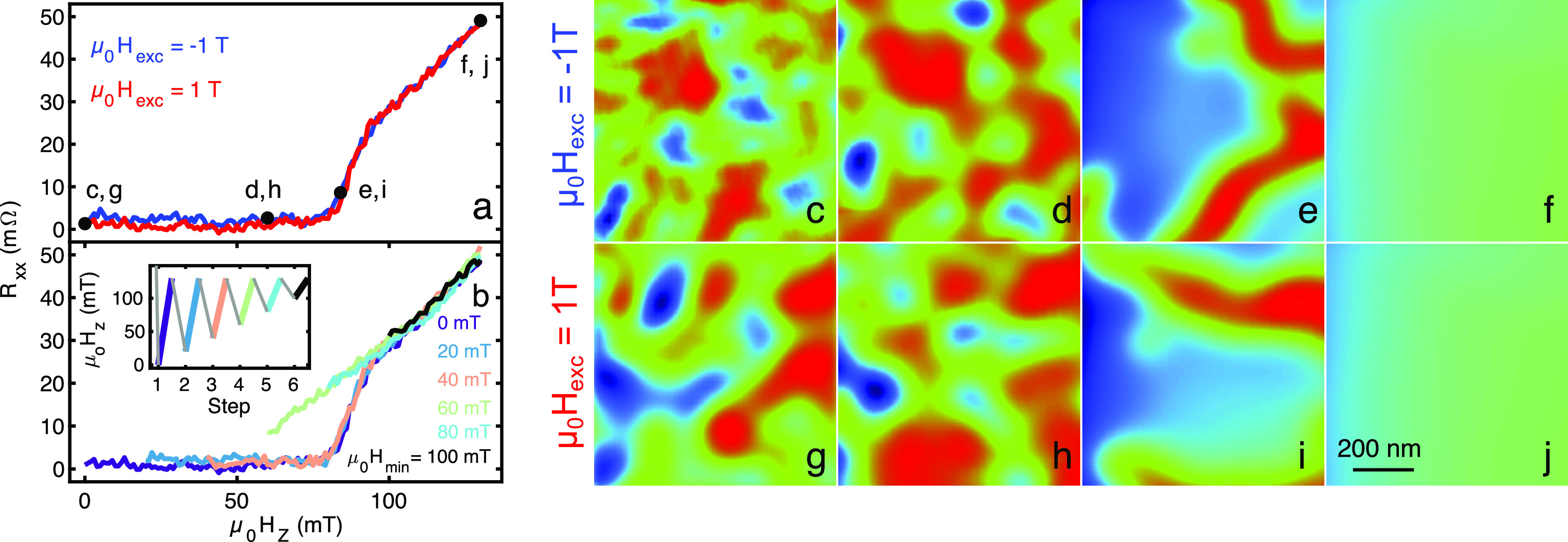
SOT images and transport of bilayer CrGeTe_3_/NbSe_2_ showing no magnetic memory at μ_0_*H*_*z*_ = 0 at 4.2K. (a) Evolution of *R*_*xx*_ as a function of the out-of-plane (OOP) field *H*_*z*_ from zero to above the saturation field *H*_s_ after an excursion to μ_0_*H*_exc_ = −1 T (blue) and μ_0_*H*_exc_ = 1 T (red). (b) Evolution of *R*_*xx*_ from μ_0_*H*_*z*_ = μ_0_*H*_min_ to μ_0_*H*_*z*_ > μ_0_*H*_s_ after applying OOP field μ_0_*H*_max_ = 130 mT saturating the sample. μ_0_*H*_min_ = 0, 20, 40, 60, 80, 100 mT. (Inset) History of *H*_*z*_ during *R*_*xx*_ measurements shown in (b) with color corresponding to the segment color. Gray segments are not shown. (c–j) Sequence of magnetic images of different states of CGT at distinct *H*_*z*_ values. Evolution from μ_0_*H*_*z*_ = 0 mT to μ_0_*H*_*z*_ > μ_0_*H*_*s*_ after *H*_exc_ = −1 T (c–f) and *H*_exc_ = 1 T **(**g–j). μ_0_*H*_*z*_ = (c, g) 0, (d, h) 60, (e, i) 80, (f, j) 130 mT. All images are 1 × 1 μm^2^, pixel size is 20 nm and acquisition time 5 min/image. The blue to red color scale represents lower and higher magnetic fields, respectively, with a shared scale of *B*_*z*_ = (c, g) 1 and (d–f, h–j) 5 mT. See Supplementary Movie 2.

It is worth noting that both the magnetic images and the transport measurement indicate magnetic hysteresis between μ_0_*H*_*z*_ = 40 mT and μ_0_*H*_*z*_ = 80 mT and that CGT demagnetizes at a positive field. Figure 1b,g shows a very similar domain structure at zero field both before and after the saturation field *H*_s_ was attained. However, the magnetic images alone cannot provide a definitive answer as to whether CGT holds any magnetization at zero field or whether CGT loses any magnetic memory in the absence of applied field. To describe the magnetic behavior near zero field, we saturated the sample by applying large opposite fields, μ_0_*H*_*z*_ = ±1 T, before changing the field back to zero and carrying out transport measurements and magnetic imaging between μ_0_*H*_*z*_ = 0 mT to μ_0_*H*_*z*_ = 130 mT (see Figure 2a). By employing this protocol, any memory that CGT might hold at zero field will be manifested as deviations in the magnetoresistance and magnetic imaging between the two excursions at either μ_0_*H*_*z*_ = +1 T or μ_0_*H*_*z*_ = −1 T. The two magnetoresistance curves presented in Figure 2a, taken after negative/positive excursions (blue/red curves) show no measurable difference between them. The magnetic images also appear to be insensitive to the change in initial conditions. Figure 2c–f show the same type of features as a function of the field as Figure 2g–j (Supplementary Movie 2). Both local (images) and global (transport) measurements show no measurable memory effect for ∼30 nm thick CGT.

The measurements shown in Figure 1 show that the ∼30 nm CGT flake retains magnetic memory and therefore is hysteretic only in the field range of μ_0_*H*_*z*_ = 40–80 mT. To verify that the sample loses memory at higher fields than zero, we ramped down the field between increasing minimal fields μ_0_*H*_min_ = 0, 20, 40, 60, 80, and 100 mT while keeping the maximum field constant and above the saturation field μ_0_*H*_max_ = 130 mT. An illustration of the measurement scheme is presented in the inset of Figure 2b. By not ramping down the field to zero, it is expected that more domains pointing with the field will act as nucleation centers to change the field at which the sample is fully magnetized.^28,29^ The magnetoresistance curves are shown in Figure 2b. The transport measurements reveal that CGT is hysteretic only when μ_0_*H*_min_ > 40 mT, i.e., CGT shows no measurable memory effect below μ_0_*H*_*z*_ = 40 mT, in excellent agreement with magnetic images that indicate 40 mT to be the demagnetization field.

The data presented in Figures 1 and 2 show two key points: that ∼30 nm CGT does not present a macroscopic finite magnetization below *H*_d_, and that the CGT flake globally demagnetizes abruptly at a field indicated by the local magnetic images (Figure 1f). Importantly, the magnetic images lend themselves to determine *H*_s_ and *H*_d_ even without the need of NbSe_2_ (or any other) metallic layer, as shown in the following.

### Thickness Dependence of CGT Magnetization

**Figure 3:**
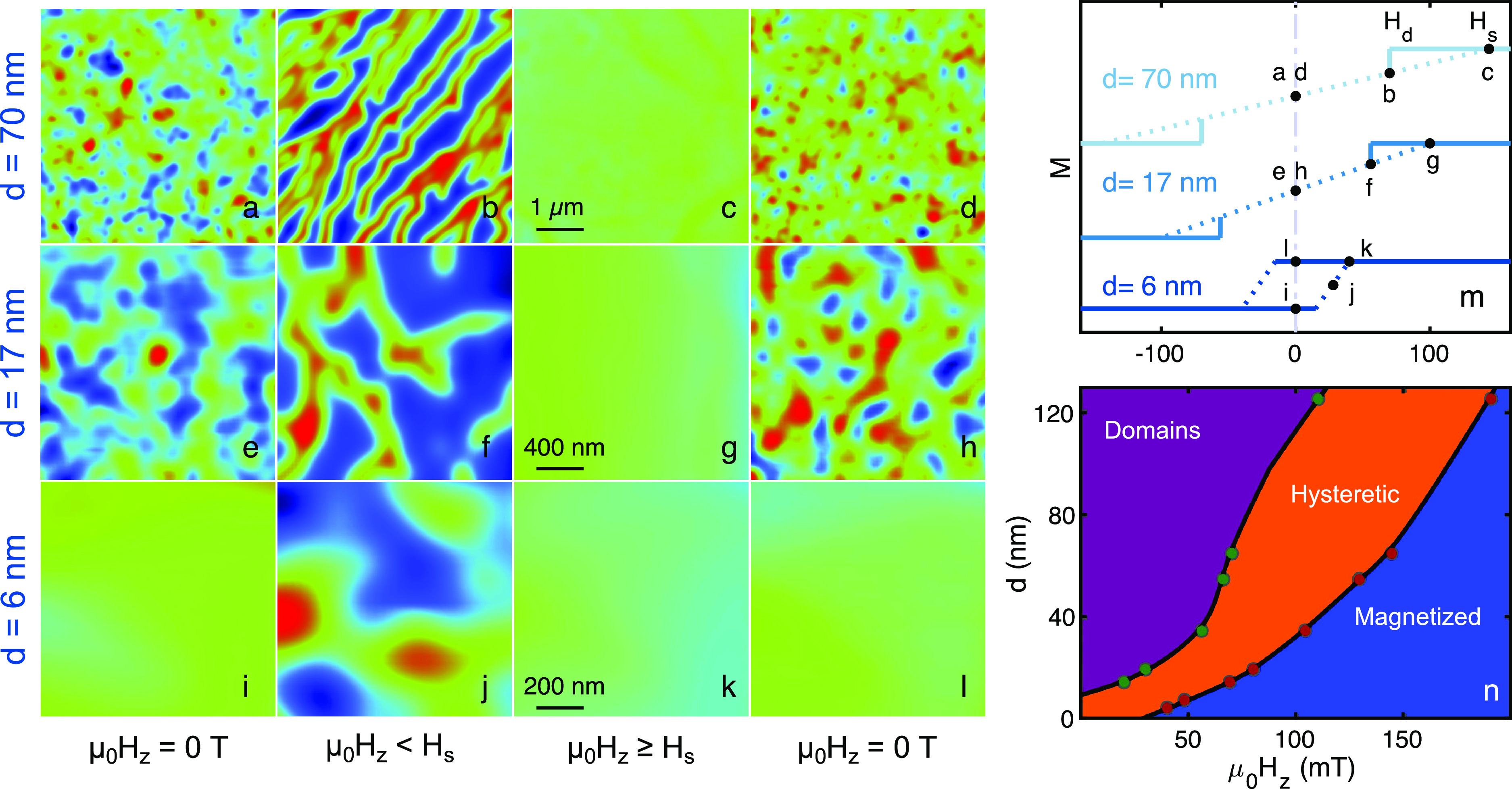
Scanning SOT microscopy images of CrGeTe_3_ for different thickness at 4.2 K. (a–l) Sequence of magnetic images *B*_*z*_(*x*, *y*) of different states of the sample at distinct values of applied out-of-plane (OOP) field μ_0_*H*_*z*_ and sample thickness *d*. (m) Sketched magnetization curves drawn from *B*_*z*_(*x*, *y*) measured on film’s parts of different *d*. Dashed lines are a guide to the eye connecting the two saturated fields. *d* = 70 nm (light blue), *d* = 17 nm (blue), *d* = 6 nm (dark blue). The fields at which the images were taken are marked with black dots. (n) A thickness-dependent magnetization-state diagram of CGT showing three states: domains (purple), hysteretic (orange), and magnetized (blue), Imaging parameters: (a–d) *d* = 70 nm, area scan 5 × 5 μm^2^, pixel size 40 nm. μ_0_*H*_*z*_ = (a) 0, (b) 115, (c) 175, (d) 0 mT. (e–h) *d* = 17 nm, area scan 2 × 2 μm^2^, pixel size 30 nm. μ_0_*H*_*z*_ = (e) 0, (f) 70, (g) 120, (h) 0 mT. (i–l) *d* = 6 nm, area scan 1 × 1 μm^2^, pixel size 30 nm. μ_0_*H*_*z*_ = (i) 0, (j) 20, (k) 120, (l) 0 mT. The blue to red color scale represents lower and higher magnetic fields, respectively, with a shared scale of *B*_*z*_ = 1 (a, c–e, h–l) and 5 (b, f, g) mT. See Supplementary Movies 3, 4, and 5, corresponding to panels c–f, g–j, and k–n, respectively. The scale bars in c, g and k apply to all images in the respective row. The *x*-axis labels of panels m and n are the same.

We now turn to the thickness dependence of the saturation and demagnetization fields. We use the SOT to image areas of distinct thickness *d* on various CGT flakes (Figure 3a–l). For areas where *d* ≳ 10 nm, the magnetic images presented in Figure 3a–h are used to find the values of *H*_s_ and *H*_d_. These values are then plotted in Figure 3m and connected to each other with a dashed line giving rise to a bowtie hysteresis loop (Figure 3m, top two sketched curves). Thinner films yield lower values of *H*_d_ and *H*_s_. For *d* ≲ 10 nm, the two hysteretic parts of the loop merge and the sample behaves like a standard ferromagnet with an open hysteresis loop (Figure 3m, bottom curve). This is seen in Figure 3i and 3l where the sample stays fully magnetized at zero field, in contrast with thicker area of the flake where the sample demagnetizes (Figure 3a,d,e,h). A comprehensive thickness dependence on sketched magnetization curves for a broad range of CGT thicknesses is plotted in Figure S3. Transport measurements similar to those shown in Figures 1 and 2 were carried out for a *d* < 10 nm CGT flake manifesting zero-field magnetization effect (See Supplementary Figure S11 and Note 4).

In Figure 3n, we summarize the values of *H*_d_ (green dots) and *H*_s_ (red dots) for all the imaged thicknesses. The lines connecting these points constitute borders between distinct magnetic states; the domains state (purple), the hysteretic state (orange), and the fully magnetized state (blue). In the domains state, CGT exhibits small magnetic domains that are insensitive to the excursion field, whereas the opposite holds for the fully magnetized region. In the hysteretic region, the sample can be either in the fully magnetized state or in the domains state depending on the applied magnetic field history.

The thickness dependence of CGT magnetization was measured here for pristine exfoliated single crystals. The recorded critical thickness for holding magnetization in zero field, ∼10 nm, is seemingly not in agreement with a few other AHE works conducted on CGT,^16,17,30^ where thicker layers of CGT seem to attain magnetic memory at zero field (finite *R*_*xx*_ at μ_0_*H*_*z*_ = 0). This might be because the above works all considered CGT proximitized to large spin orbit materials such as Pt^16^ or topological insulators (TIs) such as Bi_2_Te_3_^17^ or (Bi,Sb)_2_Te_3_.^30^ Enhanced magnetism due to hybridization of an insulating ferromagnet to a TI was also seen in a EuS/TI bilayer.^31^ Moreover, magnetic anisotropy is heavily generated due to the material spin orbit; hence, modifications of that property through proximity can adjust the magnitude of the magnetic anisotropy which, in turn, alters the magnetic properties of the ferromagnet interface.^32^ We did not observe any influence on the magnetic structure due to the superconducting proximity effect from NbSe_2_ probably because of a small spatial gap at the interface (Figure S4) hindering such a proximity effect.

### Edge Magnetization

**Figure 4:**
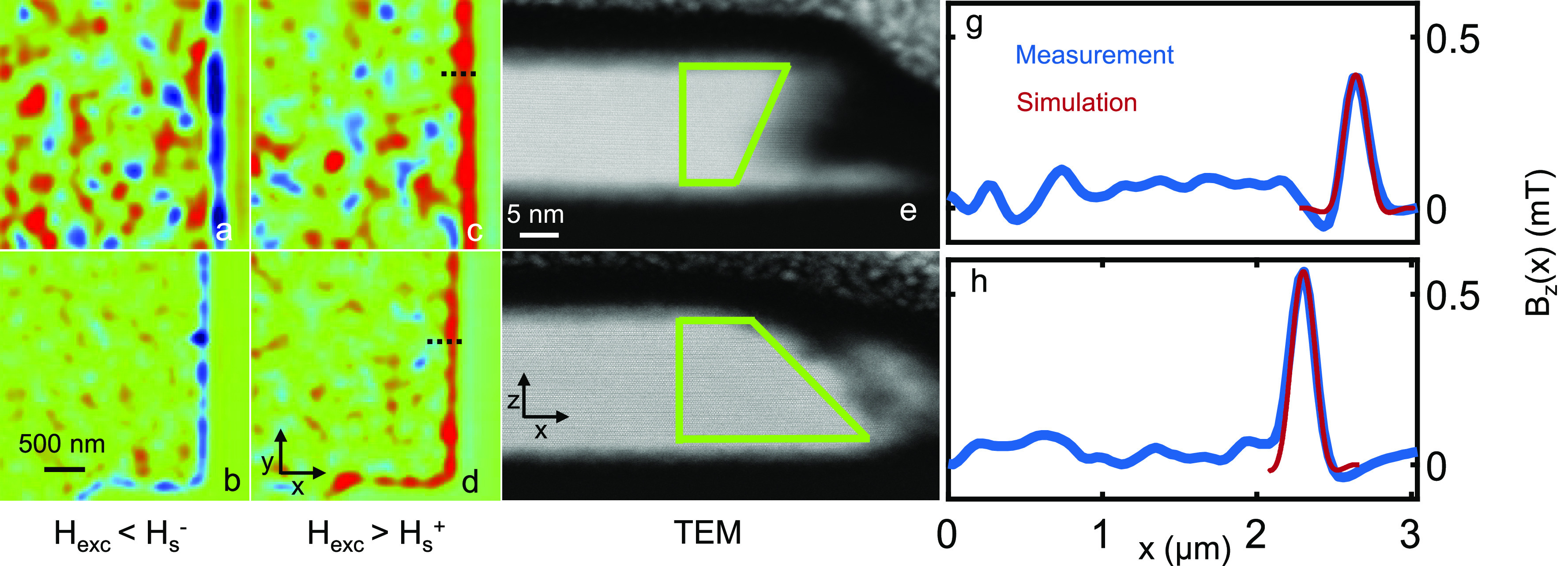
Scanning SOT microscopy images of CrGeTe_3_ flake interior and edge at zero field and STEM images of the edge. (a–d) Sequence of magnetic images acquired on two different regions of the same flake after distinct field excursions. (a, b) *H*_exc_ < *H*_s_^–^, (c, d) *H*_exc_ > *H*_s_^+^. The areas thicknesses are *d* = 17 (a, c), 24 nm (b, d). (e, f) STEM cross-sectional images measured on the black lines presented in panels c and d, respectively, lines are not to scale. (g, h) Blue lines represent the average of the local magnetic field *B*_*z*_(*x*, *y*) along the vertical (*y*) direction of panels c and d, respectively. Red lines represent the simulations of the edge magnetization stemming from magnetized edges with a trapezoid cross section, marked by green lines in panels e and f, respectively. Imaging parameters: μ_0_*H*_*z*_ = 0 mT, area scan 3 × 3 μm^2^ (pixel size (a, c) 31 and (b, d) 24 nm). The blue to red color scale represents lower and higher magnetic fields, respectively, with a shared scale for *B*_*z*_ = 1 mT.

Another possible explanation for the difference between our and previous results is that stronger magnetism is concentrated in small regions of the sample. These ferromagnetic regions might have been overrepresented in the AHE measurements carried out by other groups. With that potential contradiction in mind, we carefully imaged distinct areas of the sample. We discovered that for thick regions that show a bowtie hysteresis loop, i.e., when the flake interior breaks into domains at *H*_d_, its edge retains a magnetic memory. In Figure 4, we present two sets of images measured at μ_0_*H*_*z*_ = 0 mT after OOP field excursion to |*H*_exc_| > *H*_s_^±^. Under these conditions, domains appear in the CGT interior, but the edge clearly holds the previous magnetization direction (negative or positive, blue or red in Figure 4), determined by the polarity of previous excursion *H*_exc_, showing only small fluctuations in *B*_*z*_(*x*, *y*). The flake thicknesses presented in Figure 4 are 17 nm (Figure 4a,c) and 24 nm (Figure 4b,d). The excursion fields magnetizing the sample were: *H*_exc_ = ± 1 T (Figure 4a,c) and *H*_exc_ = 200 mT (Figure 4b,d). For samples below the critical thickness, both edge and interior behave like a hard ferromagnet and no edge magnetization is visible (Figure S8).

To try to elucidate this surprising effect, we acquired the cross-sectional scanning transmission electron microscopy (STEM) images seen in Figure 4e,f. The images reveal both the exact thickness of the measured CGT flakes and the roughness of the edge. Importantly, the edge of the sample has a tapered cross-section, thinning over a lateral distance of 10–20 nm. The average *B*_*z*_(*x*, *y*) calculated along lines in the vertical (*y*) direction as a function of *x* position in Figure 4c,d are presented as blue lines in Figure 4g,h, respectively. The average *B*_*z*_(*x*) signal peaks at ∼0.55 and ∼0.38 mT (and similar values are found for opposite excursion fields), while the inner region remains below 0.25 mT.

## Discussion

**Figure 5:**
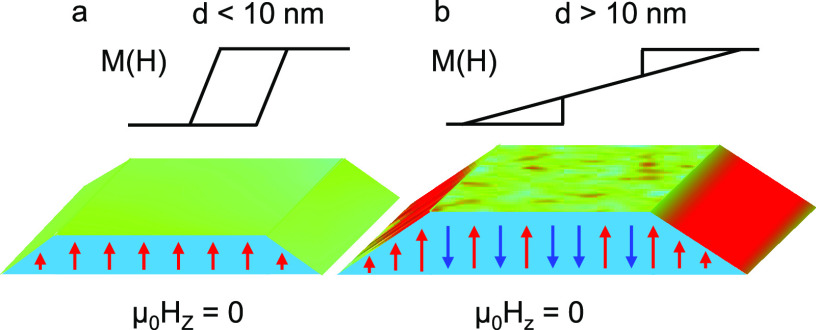
Illustration of the local magnetic structure at the edges and in the interior of CrGeTe_3_ flakes for different thicknesses. (a) Top panel, sketch of the magnetization loop. Bottom panel, local magnetic structure and the resulting out-of-plane magnetic image *B*_*z*_(*x*, *y*) at zero applied field for samples thinner than 10 nm. (b) Same as in panel a but for a sample thicker than 10 nm. The edges retain their magnetization unlike the sample’s interior. The magnetization direction of the edge at zero field depends on the field history.

Our work shows that with decreasing thickness, the saturation field *H*_s_ diminishes as well as the demagnetization field *H*_d_. This trend persists down to ∼10 nm, where for thinner flakes *H*_d_ crosses zero, thus enabling CGT to retain magnetic memory at zero field (Figure 5a,b). We note that the values of *H*_d_ and *H*_s_ were consistently observed in different areas of the same thickness irrespective of their lateral dimensions that ranged from a few micrometers to a few tens of micrometers. Finally, we also observe hard magnetism at the edges for samples above 10 nm (Figure 5b).

The vanishing remnant magnetization in zero field with increasing thickness is a phenomenon common to a number of vdW ferromagnetic materials.^33,34^ The Hamiltonian describing thin OOP magnetized ferromagnets can be written as follows:^35^
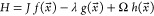
1where *J* is the exchange integral, λ is the effective magnetic anisotropy, Ω is the strength of the dipole interaction, and , , and  are the spatial functions of the magnetization. While *J* and λ correspond to local interactions stabilizing the spin magnetization, Ω is the long-range dipole interaction, making the single domain formation unstable with respect to the creation of stripe domains. Interestingly, when zero field cooling thick CGT flakes, stripe magnetization is observed (Figure S7), in agreement with the theoretical prediction in the limit where the dipolar interaction exceeds the magnetic anisotropy.^35^ In the case of strong magnetic anisotropy λ or larger exchange interaction *J*, the stripe width increases exponentially with these values,^36^ initiating an approach to the single domain phase. An accurate calculation of *J*, λ, and Ω as a function of CGT thickness was not carried out to date, though *ab initio* calculations of *J* and λ have shown qualitative agreement with experiments and were seen to change from the 2D to the bulk limit.^37^*J*, λ, and Ω are predicted to scale differently as a function of thickness,^38^ thus inducing a transition from the fragmented domain formation in the thick limit to the hard ferromagnetism in the thin limit. A similar transition was seen for FGT^34^ and was accounted for by the same model.^18^

We now discuss the edge magnetism (Figures 4 and 5b). The STEM images in Figure 4e,f reveal a variation of the flake structure on the edge, where its thickness is substantially diminished. Due to the reduced dimensionality of the edge, it is reasonable to postulate that the thinner edge behaves as the thin CGT flake (<10 nm), thereby possessing finite magnetization at zero field (Figure 5b). On the basis of this conjecture, we carried out magnetostatic simulation of the field profile generated by the thin end of the flake, depicted as a right-angled triangle cross section of area 15 × 12 nm^2^. A saturation magnetization of 3 μ_B_/Cr with a unit cell volume of 0.83 nm^3^^39^ was assumed.^26^ A convolution of the tip size with the generated stray field at the minimal possible working distance of the SOT (∼10 nm) generated an average field of 0.15–0.2 mT, smaller than the 0.38–0.55 mT measured on the edge, yet having the same direction. To better fit the measured data, the saturated section of the flake edge was increased to include a section of the thicker part of the flake as well as the thin edge, constituting trapezoid cross sections shown in Figure 4e,f. The simulated magnetism then fits well with the measured data, as can be seen by the red lines in Figure 4g,h. The simulation fitting yielded a distance of ∼100 nm between the SOT and the CGT surface, as expected. Thus, the simulation shows that the edge magnetism has a width of a few tens of nanometers. The fluctuations observed in *B*_*z*_(*x*, *y*) may be due to local variations in the effective film thickness, owing to deformations associated with the edge roughness.

The magnetization at the edge could be explained by other mechanisms, related to the in-plane dangling bounds. If such mechanisms would be dominant, then one should find magnetism also at step-edges between two terraces above the critical thickness. The absence of magnetism at such step-edges (see Figure S9) suggests that this scenario is less probable. We also did not find any preferential oxidation at the flake edge which could account for magnetization there (Figure S10). The mechanism we propose above thus appears to be a plausible one, although others could also be considered.

In conclusion, the presented study demonstrates a direct relation between the global magnetization reading of CGT by the NbSe_2_, and the local domain structure. The control of the small size domain structure can be utilized to generate highly packed magnetic memory that can be probed by GMR or superconducting wires. Small changes in thickness and edge effects can enhance the memory complexity and external field tuning ability. This effect can be also used in a double-layered device with different thicknesses of CGT, where the thick layer will act as the soft magnet and the thinner layer as the hard magnet, which may be useful for spintronics applications.

## Methods

### Sample Fabrication

Bulk NbSe_2_ was purchased from graphene HQ. We grew CGT crystals using the flux method.^40,41^ We introduced a mixture of Cr (99.99%), Ge (99.999%), and Te (99.999%) from Goodfellow in a ratio of 1:1:8 in a Canfield crucible set^42,43^ and sealed it in a quartz ampule in an argon atmosphere. We heated it to 930 °C in 12 h and slowly cooled to 500 °C in 4 days. We removed the ampule from the furnace and rapidly spun the crystals to separate the CGT crystals from excess flux. We extracted large crystals, whose size was limited by the size of the crucible. The crystals have shiny surfaces and are plate like. X-ray scattering, magnetization, and resistance versus temperature measurements will be published elsewhere and are very similar to previous reports.^26,39^

CGT and CGT/NbSe_2_ bilayer samples were fabricated using the dry transfer technique,^44^ carried out in a glovebox (argon atmosphere). NbSe_2_ and CGT flakes were cleaved using the scotch tape method, exfoliated on commercially available Gelfilm from Gelpack. For the transport measurements a NbSe_2_ flake was transferred onto prepatterned 50 nm thick Au electrodes fabricated using photolithography on a SiO_2_ substrate, and a CGT flake was subsequently transferred onto it. Both flakes were ∼30 nm thick as determined by atomic force microscopy measurements (Figures S2). The samples did not undergo heating or treatment in any solvents, deeming them pristine (other than naturally occurring oxidation upon removing the samples from the glovebox (see Supplementary Note 3 and Figure S4 and S5).

#### Transport Measurements

Transport measurements were carried out at 4.2 K inside a liquid helium dewar employing standard four-probe configuration, where the distances between the current (voltage) contacts were 15 μm (5 μm). Unless otherwise mentioned, a current bias of 250 μA was applied along the ab plane. A magnet consists of a standard SC coil was used to apply out-of-plane (OOP) magnetic fields up to ±1 T.

#### Scanning SQUID-On-Tip Microscopy

The SOT was fabricated using self-aligned three-step thermal deposition of Pb at cryogenic temperatures, as described in ref (23). Figure S1 shows the measured quantum interference pattern of one of the SOTs used for this work with an effective diameter of 155 nm and a maximum critical current of 105 μA. The asymmetric structure of the SOT gives rise to in slight shift of the interference pattern resulting a good sensitivity in zero field. All measurements were carried out at 4.2 K in low-pressure He of 1–10 mbar.

## Supporting Information Available

Additional experimental details and discussion such as SOT images at ZFC and edge of 6 nm CGT flake, magneto-transport measurements of CGT/NbSe_2_ with *d* < 10 nm, movies of protocols present in the manuscript, optical, atomic force microscopy, STEM and EDX measurements of the main CGT flake used for the thickness dependence measurements, and information on the SOT parameters. (PDF)
Supplementary Movies 1 and 2: Magnetic domains evolution imaged with the SOT and longitudinal resistance from zero field to the saturated state and back to zero in CrGeTe_3_ and in 30 nm CrGeTe_3_ after excursion of μ_0_*H*_exc_ = ±1 T, respectively (MP4, MP4)
Supplementary Movies 3–5: Magnetic domains evolution imaged with the SOT between saturated states of 65 nm CrGeTe_3_ thin film, between saturated states of 25 nm CrGeTe_3_ thin film, and between saturated states through an intermediate domain phase in 6 nm CrGeTe_3_ thin film, respectively (MP4, MP4, MP4)

## References

[ref1] Dean C. R.; Young A. F.; Meric I.; Lee C.; Wang L.; Sorgenfrei S.; Watanabe K.; Taniguchi T.; Kim P.; Shepard K. L.; Hone J. Boron Nitride Substrates for High-Quality Graphene Electronics. Nat. Nanotechnol. 2010, 5 (10), 722–726. 10.1038/nnano.2010.172. 20729834

[ref2] Ponomarenko L. A.; Geim A. K.; Zhukov A. A.; Jalil R.; Morozov S. V.; Novoselov K. S.; Grigorieva I. V.; Hill E. H.; Cheianov V. V.; Fal’ko V. I.; Watanabe K.; Taniguchi T.; Gorbachev R. V. Tunable Metal–Insulator Transition in Double-Layer Graphene Heterostructures. Nature Phys. 2011, 7 (12), 958–961. 10.1038/nphys2114.

[ref3] Xia F.; Mueller T.; Lin Y.; Valdes-Garcia A.; Avouris P. Ultrafast Graphene Photodetector. Nat. Nanotechnol. 2009, 4 (12), 839–843. 10.1038/nnano.2009.292. 19893532

[ref4] Lin Z.; Huang Y.; Duan X. Van Der Waals Thin-Film Electronics. Nat. Electron 2019, 2 (9), 378–388. 10.1038/s41928-019-0301-7.

[ref5] Li H.; Ruan S.; Zeng Y.-J. Intrinsic Van Der Waals Magnetic Materials from Bulk to the 2D Limit: New Frontiers of Spintronics. Adv. Mater. 2019, 31 (27), 1900065 10.1002/adma.201900065. 31069896

[ref6] Han W.; Kawakami R. K.; Gmitra M.; Fabian J. Graphene Spintronics. Nat. Nanotechnol. 2014, 9 (10), 794–807. 10.1038/nnano.2014.214. 25286274

[ref7] Sierra J. F.; Fabian J.; Kawakami R. K.; Roche S.; Valenzuela S. O. van der Waals Heterostructures for Spintronics and Opto-Spintronics. Nat. Nanotechnol. 2021, 16, 856–868. 10.1038/s41565-021-00936-x. 34282312

[ref8] Gong C.; Li L.; Li Z.; Ji H.; Stern A.; Xia Y.; Cao T.; Bao W.; Wang C.; Wang Y.; Qiu Z. Q.; Cava R. J.; Louie S. G.; Xia J.; Zhang X. Discovery of Intrinsic Ferromagnetism in Two-Dimensional van Der Waals Crystals. Nature 2017, 546 (7657), 265–269. 10.1038/nature22060. 28445468

[ref9] Fei Z.; Huang B.; Malinowski P.; Wang W.; Song T.; Sanchez J.; Yao W.; Xiao D.; Zhu X.; May A. F.; Wu W.; Cobden D. H.; Chu J.-H.; Xu X. Two-Dimensional Itinerant Ferromagnetism in Atomically Thin Fe3GeTe2. Nat. Mater. 2018, 17 (9), 778–782. 10.1038/s41563-018-0149-7. 30104669

[ref10] Huang B.; Clark G.; Navarro-Moratalla E.; Klein D. R.; Cheng R.; Seyler K. L.; Zhong D.; Schmidgall E.; McGuire M. A.; Cobden D. H.; Yao W.; Xiao D.; Jarillo-Herrero P.; Xu X. Layer-Dependent Ferromagnetism in a van Der Waals Crystal down to the Monolayer Limit. Nature 2017, 546 (7657), 270–273. 10.1038/nature22391. 28593970

[ref11] Mermin N. D.; Wagner H. Absence of Ferromagnetism or Antiferromagnetism in One- or Two-Dimensional Isotropic Heisenberg Models. Phys. Rev. Lett. 1966, 17 (22), 1133–1136. 10.1103/PhysRevLett.17.1133.

[ref12] León-Brito N.; Bauer E. D.; Ronning F.; Thompson J. D.; Movshovich R. Magnetic Microstructure and Magnetic Properties of Uniaxial Itinerant Ferromagnet Fe3GeTe2. J. Appl. Phys. 2016, 120 (8), 083903 10.1063/1.4961592.

[ref13] Zhang X.; Zhao Y.; Song Q.; Jia S.; Shi J.; Han W. Magnetic Anisotropy of the Single-Crystalline Ferromagnetic Insulator Cr2Ge2Te6. Jpn. J. Appl. Phys. 2016, 55 (3), 033001 10.7567/JJAP.55.033001.

[ref14] Singh C. K.; Kabir M. Long-Range Anisotropic Heisenberg Ferromagnets and Electrically Tunable Ordering. Phys. Rev. B 2021, 103 (21), 214411 10.1103/PhysRevB.103.214411.

[ref15] Liu S.; Yuan X.; Zou Y.; Sheng Y.; Huang C.; Zhang E.; Ling J.; Liu Y.; Wang W.; Zhang C.; Zou J.; Wang K.; Xiu F. Wafer-Scale Two-Dimensional Ferromagnetic Fe3GeTe2 Thin Films Grown by Molecular Beam Epitaxy. npj 2D Mater. Appl. 2017, 1 (1), 1–7. 10.1038/s41699-017-0033-3.

[ref16] Lohmann M.; Su T.; Niu B.; Hou Y.; Alghamdi M.; Aldosary M.; Xing W.; Zhong J.; Jia S.; Han W.; Wu R.; Cui Y.-T.; Shi J. Probing Magnetism in Insulating Cr2Ge2Te6 by Induced Anomalous Hall Effect in Pt. Nano Lett. 2019, 19 (4), 2397–2403. 10.1021/acs.nanolett.8b05121. 30823703

[ref17] Alegria L. D.; Ji H.; Yao N.; Clarke J. J.; Cava R. J.; Petta J. R. Large Anomalous Hall Effect in Ferromagnetic Insulator-Topological Insulator Heterostructures. Appl. Phys. Lett. 2014, 105 (5), 053512 10.1063/1.4892353.

[ref18] Li Q.; Yang M.; Gong C.; Chopdekar R. V.; N’Diaye A. T.; Turner J.; Chen G.; Scholl A.; Shafer P.; Arenholz E.; Schmid A. K.; Wang S.; Liu K.; Gao N.; Admasu A. S.; Cheong S.-W.; Hwang C.; Li J.; Wang F.; Zhang X.; Qiu Z. Patterning-Induced Ferromagnetism of Fe3GeTe2 van Der Waals Materials beyond Room Temperature. Nano Lett. 2018, 18 (9), 5974–5980. 10.1021/acs.nanolett.8b02806. 30114354

[ref19] Thiel L.; Wang Z.; Tschudin M. A.; Rohner D.; Gutiérrez-Lezama I.; Ubrig N.; Gibertini M.; Giannini E.; Morpurgo A. F.; Maletinsky P. Probing Magnetism in 2D Materials at the Nanoscale with Single-Spin Microscopy. Science 2019, 364 (6444), 973–976. 10.1126/science.aav6926. 31023891

[ref20] Uri A.; Kim Y.; Bagani K.; Lewandowski C. K.; Grover S.; Auerbach N.; Lachman E. O.; Myasoedov Y.; Taniguchi T.; Watanabe K.; Smet J.; Zeldov E. Nanoscale Imaging of Equilibrium Quantum Hall Edge Currents and of the Magnetic Monopole Response in Graphene. Nat. Phys. 2020, 16 (2), 164–170. 10.1038/s41567-019-0713-3.

[ref21] Uri A.; Grover S.; Cao Y.; Crosse J. A.; Bagani K.; Rodan-Legrain D.; Myasoedov Y.; Watanabe K.; Taniguchi T.; Moon P.; Koshino M.; Jarillo-Herrero P.; Zeldov E. Mapping the Twist-Angle Disorder and Landau Levels in Magic-Angle Graphene. Nature 2020, 581 (7806), 47–52. 10.1038/s41586-020-2255-3. 32376964

[ref22] Noah A.; Toric F.; Feld T. D.; Zissman G.; Gutfreund A.; Tsruya D.; Devidas T. R.; Alpern H.; Steinberg H.; Huber M. E.; Analytis J. G.; Gazit S.; Lachman E.; Anahory Y. Hidden Spin-Texture at Topological Domain Walls Drive Exchange Bias in a Weyl Semimetal. arXiv (Materials Science), January 27, 2021, 2101.11639, ver. 1. DOI: 10.48550/arXiv.2101.11639 (accessed 03/03/2022).

[ref23] Vasyukov D.; Anahory Y.; Embon L.; Halbertal D.; Cuppens J.; Neeman L.; Finkler A.; Segev Y.; Myasoedov Y.; Rappaport M. L.; Huber M. E.; Zeldov E. A Scanning Superconducting Quantum Interference Device with Single Electron Spin Sensitivity. Nat. Nanotechnol. 2013, 8 (9), 639–644. 10.1038/nnano.2013.169. 23995454

[ref24] Anahory Y.; Naren H. R.; Lachman E. O.; Buhbut Sinai S.; Uri A.; Embon L.; Yaakobi E.; Myasoedov Y.; Huber M. E.; Klajn R.; Zeldov E. SQUID-on-Tip with Single-Electron Spin Sensitivity for High-Field and Ultra-Low Temperature Nanomagnetic Imaging. Nanoscale 2020, 12 (5), 3174–3182. 10.1039/C9NR08578E. 31967152

[ref25] Nutting D.; Withers F. Flux Pinning in NbSe2 - CrGeTe3 Heterostructures. Physica C: Superconductivity and its Applications 2021, 581, 1353803 10.1016/j.physc.2020.1353803.

[ref26] Ji H.; Stokes R. A.; Alegria L. D.; Blomberg E. C.; Tanatar M. A.; Reijnders A.; Schoop L. M.; Liang T.; Prozorov R.; Burch K. S.; Ong N. P.; Petta J. R.; Cava R. J. A Ferromagnetic Insulating Substrate for the Epitaxial Growth of Topological Insulators. J. Appl. Phys. 2013, 114 (11), 114907 10.1063/1.4822092.

[ref27] Tinkham M. Introduction to Superconductivity, 2nd ed.; Dover Publications: Mineola, NY, 2004.

[ref28] Windsor Y. W.; Gerber A.; Karpovski M. Dynamics of Successive Minor Hysteresis Loops. Phys. Rev. B 2012, 85 (6), 064409 10.1103/PhysRevB.85.064409.

[ref29] Windsor Y. W.; Gerber A.; Korenblit I. Ya.; Karpovski M. Time Dependence of Magnetization Reversal When Beginning with Pre-Existing Nucleation Sites. J. Appl. Phys. 2013, 113 (22), 223902 10.1063/1.4809762.

[ref30] Mogi M.; Tsukazaki A.; Kaneko Y.; Yoshimi R.; Takahashi K. S.; Kawasaki M.; Tokura Y. Ferromagnetic Insulator Cr2Ge2Te6 Thin Films with Perpendicular Remanence. APL Materials 2018, 6 (9), 091104 10.1063/1.5046166.

[ref31] Wei P.; Katmis F.; Assaf B. A.; Steinberg H.; Jarillo-Herrero P.; Heiman D.; Moodera J. S. Exchange-Coupling-Induced Symmetry Breaking in Topological Insulators. Phys. Rev. Lett. 2013, 110 (18), 186807 10.1103/PhysRevLett.110.186807. 23683236

[ref32] Gruszecki P.; Banerjee C.; Mruczkiewicz M.; Hellwig O.; Barman A.; Krawczyk M. Chapter Two - The Influence of the Internal Domain Wall Structure on Spin Wave Band Structure in Periodic Magnetic Stripe Domain Patterns. Solid State Phys. 2019, 70, 79–132. 10.1016/bs.ssp.2019.09.003.

[ref33] Liu Y.; Wu L.; Tong X.; Li J.; Tao J.; Zhu Y.; Petrovic C. Thickness-Dependent Magnetic Order in CrI3 Single Crystals. Sci. Rep 2019, 9 (1), 13599 10.1038/s41598-019-50000-x. 31537855 PMC6753156

[ref34] Tan C.; Lee J.; Jung S.-G.; Park T.; Albarakati S.; Partridge J.; Field M. R.; McCulloch D. G.; Wang L.; Lee C. Hard Magnetic Properties in Nanoflake van Der Waals Fe3GeTe2. Nat. Commun. 2018, 9 (1), 1554 10.1038/s41467-018-04018-w. 29674662 PMC5908800

[ref35] Kashuba A.; Pokrovsky V. L. Stripe Domain Structures in a Thin Ferromagnetic Film. Phys. Rev. Lett. 1993, 70 (20), 3155–3158. 10.1103/PhysRevLett.70.3155. 10053789

[ref36] Wu Y. Z.; Won C.; Scholl A.; Doran A.; Zhao H. W.; Jin X. F.; Qiu Z. Q. Magnetic Stripe Domains in Coupled Magnetic Sandwiches. Phys. Rev. Lett. 2004, 93 (11), 117205 10.1103/PhysRevLett.93.117205. 15447377

[ref37] Fang Y.; Wu S.; Zhu Z.-Z.; Guo G.-Y. Large Magneto-Optical Effects and Magnetic Anisotropy Energy in Two-Dimensional Cr2Ge2Te6. Phys. Rev. B 2018, 98 (12), 125416 10.1103/PhysRevB.98.125416.

[ref38] Won C.; Wu Y. Z.; Choi J.; Kim W.; Scholl A.; Doran A.; Owens T.; Wu J.; Jin X. F.; Zhao H. W.; Qiu Z. Q. Magnetic Stripe Melting at the Spin Reorientation Transition in Fe/Ni/Cu(001). Phys. Rev. B 2005, 71 (22), 224429 10.1103/PhysRevB.71.224429.

[ref39] Carteaux V.; Brunet D.; Ouvrard G.; Andre G. Crystallographic, Magnetic and Electronic Structures of a New Layered Ferromagnetic Compound Cr2Ge2Te6. J. Phys.: Condens. Matter 1995, 7 (1), 69–87. 10.1088/0953-8984/7/1/008.

[ref40] Canfield P. C.; Fisk Z. Growth of Single Crystals from Metallic Fluxes. null 1992, 65 (6), 1117–1123. 10.1080/13642819208215073.

[ref41] Canfield P. C. Solution Growth of Intermetallic Single Crystals: A Beginner’s Guide. Book Ser. Complex Met. Alloys 2009, 2, 93–111. 10.1142/9789814261647_0002.

[ref42] Canfield P. C.; Kong T.; Kaluarachchi U. S.; Jo N. H. Use of Frit-Disc Crucibles for Routine and Exploratory Solution Growth of Single Crystalline Samples. Philos. Mag. 2016, 96 (1), 84–92. 10.1080/14786435.2015.1122248.

[ref43] Canfield P. C. New Materials Physics. Rep. Prog. Phys. 2020, 83 (1), 016501 10.1088/1361-6633/ab514b. 31652428

[ref44] Castellanos-Gomez A.; Buscema M.; Molenaar R.; Singh V.; Janssen L.; van der Zant H. S. J.; Steele G. A. Deterministic Transfer of Two-Dimensional Materials by All-Dry Viscoelastic Stamping. 2D Mater. 2014, 1 (1), 011002 10.1088/2053-1583/1/1/011002.

