# Supplementary material for: Interior and Edge Magnetization in Thin Exfoliated CrGeTe3 Films
Source: Nano Lett. 2022 Mar 10;22(7):3165–72. doi: 10.1021/acs.nanolett.1c04665 (PMC9011403; doi:10.1021/acs.nanolett.1c04665)
Supplement: Supplementary file 1 — nl1c04665_si_001.pdf [file nl1c04665_si_001.pdf]

Supplementary material

Interior and edge magnetization in thin exfoliated $CrGeTe_3$ films

Avia Noah[1], Hen Alpern[1,2**], Sourabh Singh[1], Alon Gutfreund[1], Gilad Zisman[1], Tomer D. Feld[1], Atzmon Vakahi[3], Sergei Remennik[3], Yossi Paltiel[2], Martin Emile Huber[4], Victor Barrena[5], Hermann Suderow[5], Hadar Steinberg[1], Oded Millo[1], and Yonathan Anahory***[1]*

[1]The Racah Institute of Physics, The Hebrew University, Jerusalem, 91904, Israel
[2]Department of Applied Physics, The Hebrew University of Jerusalem, Jerusalem 91904, Israel
[3]Center for Nanoscience and Nanotechnology, Hebrew University of Jerusalem, Jerusalem, 91904, Israel
[4]Departments of Physics and Electrical Engineering, University of Colorado Denver, Denver, CO 80217, USA
[5]Laboratorio de Bajas Temperaturas, Unidad Asociada UAM/CSIC, Departamento de Física de la Materia Condensada, Instituto Nicolás Cabrera and Condensed Matter Physics Center, Universidad Autónoma de Madrid, E-28049 Madrid, Spain

Corresponding authors: *avia.noah@mail.huji.ac.il, ** alprnhen@gmail.com,
*** yonathan.anahory@mail.huji.ac.il

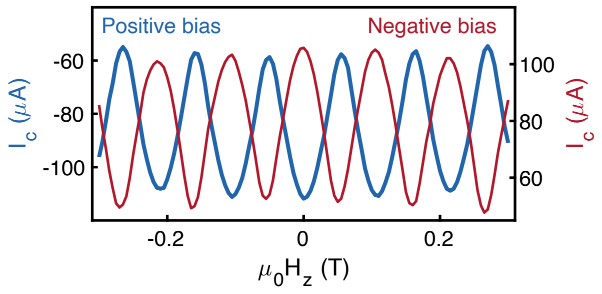

Supplementary Figure S1. Quantum interference pattern of the SQUID-on-tip (SOT). The critical current I_c of one of the SOT's used in this work as a function of the applied out-of-Plane field H_z. Blue: Positive bias, red: Negative bias. The period of 105 mT of the quantum interference provides an effective diameter of 155 nm of the SOT.

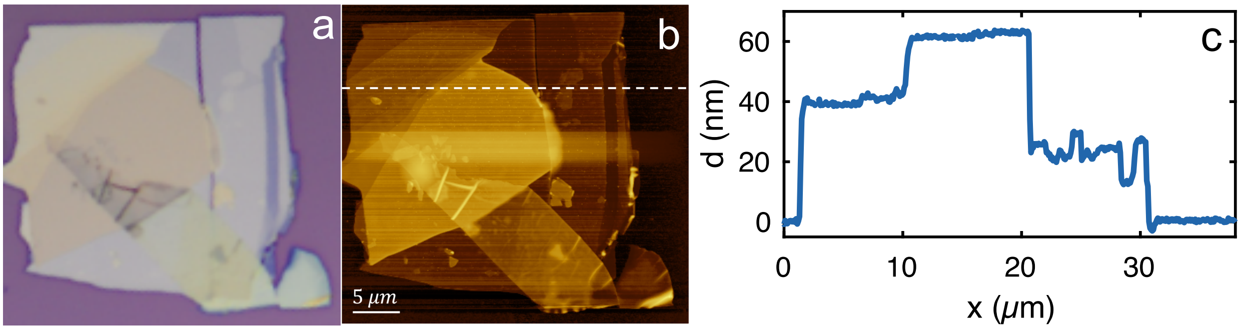

Supplementary Figure S2. AFM and optical thickness characterization. (a) Optical image of an exfoliated CGT flake on a SiO_2 substrate. The CGT flake contains varying thicknesses with correlated colors ranging from light purple (thinnest) to yellow (thickest). (b) A topography image of the flake seen in panel a, taken by an AFM using the tapping mode. (c) A topography cross section taken along the dashed line in panel b, sowing areas with distinct (uniform) thicknesses.

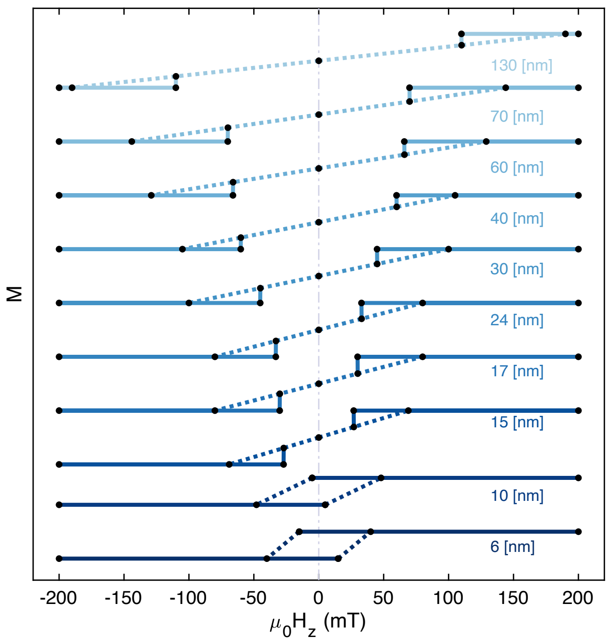

Supplementary Figure S3. Transport of bilayer CGT/$NbSe_2$ at different thickness at 4.2 K. Sketched magnetization curves drawn from $B_z(x, y)$ measured on film's parts of different d. Dashed lines are a guide to the eye connecting the two saturated fields. Thickness from bottom to top $d =$ 6, 10 ,15, 17, 24, 30, 40, 60, 70, 130 nm

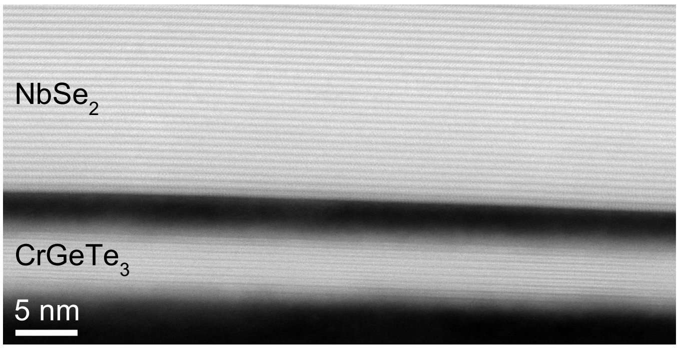

Supplementary Figure S4. High angle annular dark-HAADF image of the heterostructure in the sacrificial device. The CGT layers are seen to be separated by a gap from the $NbSe_2$ layers.

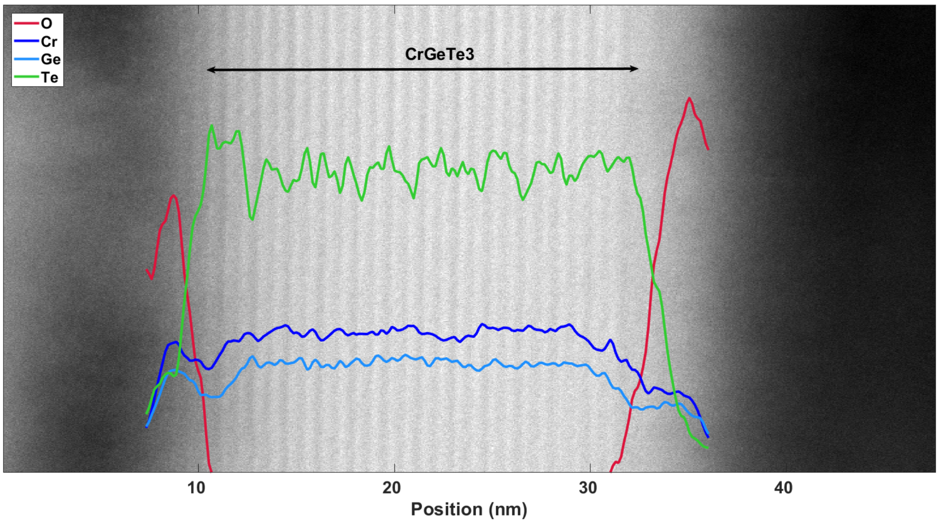

Supplementary Figure S5. EDS line scan. Showing the relative amount of Cr, Ge, Te and O in a cross section of the device, in arbitrary units. The results are superimposed on a high-angle annular dark field (HAADF) image of the same cross section.

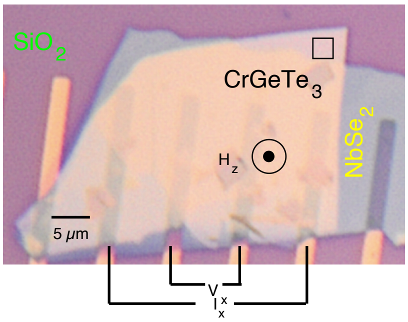

Supplementary Figure 6. Optical image of the bilayer and the Au electrodes used for the transport measurements presented in figure 1 and 2 of the main text. The black square corresponds to the region image in Fig. 1 b-g and Fig. 2. c-j of the main text.

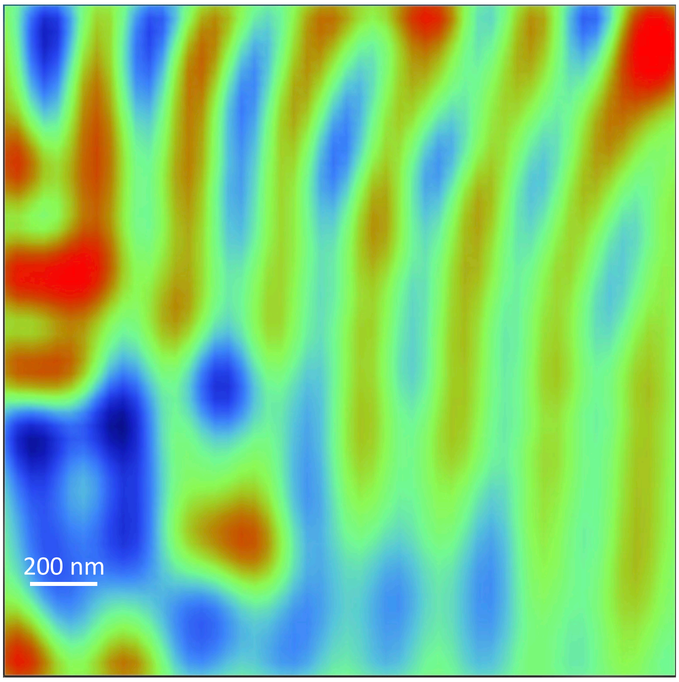

Supplementary Figure S7. SQUID-on-tip image of 130 nm $CrGeTe_3$ after zero field cooling (SOT). Stripe magnetization with zero net magnetization in agreement with the theoretical prediction in the limit where the dipolar interaction exceeds the magnetic anisotropy. area scan 2x2 μm², pixel size 52 nm. The blue to red color scale represents lower and higher magnetic field respectively.

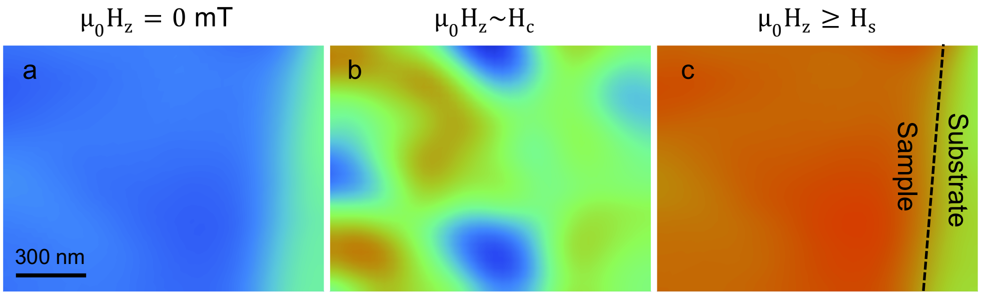

Supplementary Figure S8. Scanning SOT microscopy images of a 6 nm thick $CrGeTe_3$ flake interior. (**a**-**c**) Time sequence of magnetic images at distinct applied fields. (**a**) At zero field, after an excursion at $\mu_0 H_z = -200$ mT (larger than H_s). (**b**) Near the coercive field $H_c = 18$ mT (**c**) at 120 mT, above the saturation filed, where the sample is fully magnetized in the other direction. Imaging parameters: area scan 1.5×1 μm², pixel size 31 nm. The blue to red color scale represents lower and higher magnetic fields, respectively, while the green color represents the applied field. The full color scale is $B_z = $ 1 mT in all images.

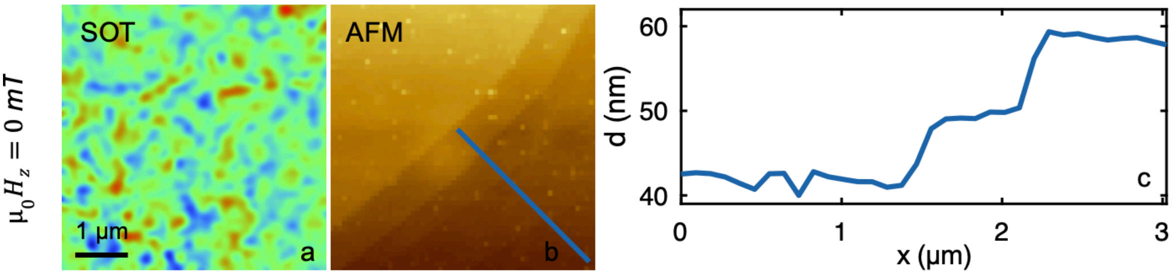

Supplementary Figure S9. SOT image of $CrGeTe_3$ flake with a corresponding AFM image. (**a**) SOT magnetic image at $\mu_0 H_z = 0$ mT. The magnetic domains evolve with no relation to the step-edges. (**b)** AFM image of the same scanning area resolving steps of $\sim$ 10 nm. (**c**) Height profile measured along the blue line presented in **b**. Image parameters: area scan 5×5 μm^2, pixel size 26 nm. The blue to red color scale represents lower and higher magnetic field, respectively, with a scale of $B_z = 1$ mT.

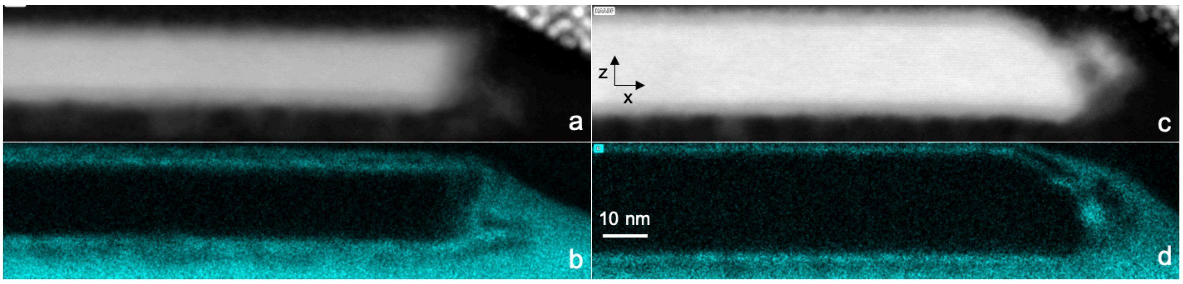

Supplementary Figure S10. EDX and STEM of 17 and 24 nm flake edges. (**a-b**) STEM cross-sectional images measured on the black lines presented in **Fig. 4 c,d**. **(c-d)** EDX measurements of the same cross-sections showing similar levels of oxidation on the edge and surface.

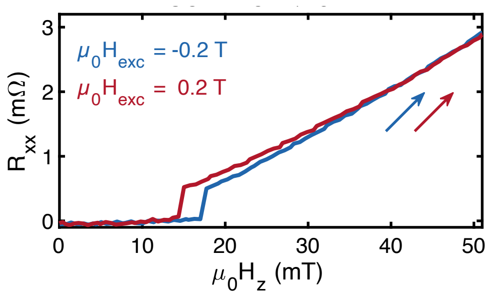

Supplementary Figure S11. R_{xx} magneto-transport measurements of bilayer $CrGeTe_3/NbSe_2$ showing magnetic memory at $\mu_0 H_z = 0$ for CGT of thickness $d < 10$ nm. R_{xx} as a function of the out-of-plane (OOP) field H_z from zero to above the saturation field H_s after different excursions at $\mu_0 H_{ex} = -0.2$ T and $\mu_0 H_{ex} = +0.2$ T, blue and red curves, respectively.

Supplementary Note 1: SOT fabrication and characterization

Supplementary figure 1 shows the quantum interference pattern typical for a SQUID. The SOT response to magnetic field is linked to the derivative of that pattern. To get good images, the SOT has to be field biased in a region where the interference pattern is linear, and one must avoid the regions where the response is zero (blind spots). The field period of this pattern is determined by the SQUID loop diameter so that each period represents a magnetic flux equal to the quantum of flux $\phi_0 = h/2e \approx 20.67$ Gμm^2. This means that a smaller SQUID loop will yield a large field period with larger linear regions but with larger blind spots. Depending on the experimental requirements, optimal SQUID loop size is chosen. To mitigate the effect of blind spots, we use the fact that our SOTs are often composed of asymmetric junctions. This makes the interference pattern to have a different field offset for a different direction of the current running through the SQUID (Supplementary Figure 1, blue and red curves).

The magnetic length scale in $CrGeTe_3$ is on the order 100 nm or smaller. That implies a SQUID loop with a diameter below 100 nm. However, having a SQUID with a loop too small, of 50 nm for example, would yield a blind spot of +/- 0.4 T around zero field. That would be problematic to get good images below the saturation field (0.13 T). For this reason, all the SOTs used in this work have a SQUID loop of about 150 to 180 nm.

Supplementary Note 2: Atomic force microscopy (AFM) measurements

Topography measurements of CGT flakes exfoliated on Si-SiO_2 substrates were conducted to determine the thickness of the various areas measured by SOT. To that end a Ntegra modular apparatus (NT-MDT) was employed using the tapping method. Only areas showing thickness uniformity were used during the SOT characterization. An AFM (as well as an optical image) of one of the CGT flakes used in this work is shown in Supplementary Figure 2.

Supplementary Note 3: STEM images of CGT with thickness analysis.

To investigate the engagement between the $NbSe_2$ and $CrGeTe_3$ (CGT) we performed cross-section scanning transmission electron microscopy (STEM) and energy-dispersive spectroscopy (EDS) on the heterostructure device. High angle annular dark-field (HAADF) STEM image of a sacrificial device is shown in Supplementary Figure 4 which depicts a gap between the CGT and the $NbSe_2$. An average spacing of roughly 4 nm between the layers is seen in the figure. To understand the stoichiometry of the flakes EDS was performed and it reveals formation of an oxide layer on both sides of the flakes. Traces of Silicon and Carbon (not shown) were also observed in the EDS measurements which seem to originate because of the organic residue from the PDMS used during the exfoliation process.

To determine the thickness of thin CGT flakes we took the cross-sectional STEM images shown in Supplementary Figures 5 where individual layers of CGT can be seen. There is a formation of oxide layer on both sides of the flakes as seen from the fuzzy layers on top and bottom of the flake. The EDS line scan along the black arrow confirm the presence of Oxygen on the top and bottom of CGT flakes.

Supplementary Note 4: Transport of a CGT/$NbSe_2$ bilayer with $d < 10$ nm

We measured a CGT/$NbSe_2$ bilayer similar to the ones presented in Figures 1 and 2, but with a CGT layer below the critical thickness. The results are shown in Figure S11. First, we see a difference in $R_{xx}(H)$ when the sample was initialized at -0.2 T and +0.2 T. This clearly shows the presence of a global zero-field memory which is not observed for thicker samples (Figure 1 and 2). Second, we observe a raise in the field at which the voltage appears when the CGT was previously magnetized at $\mu_0 H_{ex} = -0.2$ T, opposite to the measurement field. This suggests that the sample was fully magnetized opposite to the field therefore reducing the field on the $NbSe_2$ and therefore the amount of vortices. Third, the two $R_{xx}(H)$ curves overlap above 35 mT, no matter what the previous history was. This suggests that the magnetic state of the CGT is fully polarized above that field which is consistent with the saturation field stated in Figure 3. Finally, around the coercive field, between ~20 to ~30 mT, the resistance after excursion at opposite field (-0.2 T, blue curve) is lower than that after excursion at 0.2 T (red curve), suggesting an enhancement of pinning due to the emergence of domains during the magnetization reversal process that takes place only when measuring the blue curve.

Supplementary Movie 1:

Movie of magnetic domains evolution imaged with the SOT and longitudinal resistance (R_{xx}) from zero field to the saturated state and back to zero in $CrGeTe_3$. $\mu_0 H_z$ was ramped up from 0 to 130 mT (blue curve) and then, promptly, ramped down back to 0 (red curve). Yellow dot represents the field at which the image of the out-of-plane component of the magnetic field $B_z(x,y)$ was taken. Magnetic features of $\sim$100 nm exhibiting a magnetic contrast ($\sim$5 mT) were observed. By increasing H_z, the domains that are anti-aligned with the field shrink while the ones that are parallel growing. From $\mu_0 H_z \sim 100$ mT, the magnetic contrast drops below 1 mT and the $B_z(x,y)$ stops evolving on that scale. By decreasing the field, the sample's magnetic images remain featureless down to $\mu_0 H_z = 40$ mT, where magnetic domains appear at once. A clear hysteresis is evident in the field range values between $\mu_0 H_z = 40$ mT and $\mu_0 H_z = 80$ mT, where a switching between the dissipationless and voltage states occurs. The frame size was 1×1 μm^2; pixel 20 nm, acquisition time was 5 min/image and H_z was increased in 5 mT step. The blue to red color scale represents lower and higher magnetic field, respectively; when high magnetic contrast appears, the color scale is intentionally saturated at the edge for clarity. Selected frames from the movie are shown Fig. 1 of the main text.

Supplementary Movie 2:

Movie of magnetic domains evolution imaged with the SOT and longitudinal resistance (R_{xx}) from zero field to the saturated state in 30 nm $CrGeTe_3$ after excursion of $\mu_0 H_{exc} = \pm 1$ T. $\mu_0 H_z$ was ramped up from 0 to 130 mT. The last dot represents the field at which the image of the out-of-plane component of the magnetic field $B_z(x,y)$ was taken. Magnetic features of $\sim$ 100 nm exhibiting a magnetic contrast ($\sim$5 mT) were observed. By increasing H_z, the domains that are anti-aligned with the field shrink while the ones that are parallel growing. From $\mu_0 H_z \sim 100$ mT, the magnetic contrast drops below 1 mT and the $B_z(x,y)$ stops evolving on that scale. The same evolution both by images and transport were observed resolving zero magnetization at zero field. The frame size was 1×1 μm^2; pixel 20 nm, acquisition time was 5 min/image and H_z was increased in 5 mT step. The blue to red color scale represents lower and higher magnetic field, respectively; when high magnetic contrast appears, the color scale is intentionally saturated at the edge for clarity. Selected frames from the movie are shown Fig. 2 of the main text.

Supplementary Movie 3:

Movie of magnetic domains evolution imaged with the SOT between saturated states of 65 nm $CrGeTe_3$ thin film. $\mu_0 H_z$ was ramped up from 0 to 200 mT, then ramped down to -200 mT and back to zero. Magnetic features of ~ 100 nm were observed. By increasing H_z, the domains that are anti-aligned with the field shrink while the ones that are parallel growing. From $\mu_0 H_z \sim 145$ mT, the magnetic contrast drops below 1 mT and the $B_z(x,y)$ stops evolving on that scale. By decreasing the field, the sample's magnetic images remain featureless down to $\mu_0 H_z = 70$ mT, where magnetic domains appear at once. The same behavior was observed for the negative sweep. The frame size was 5×5 μm^2; pixel 40 nm, acquisition time was 5 min/image and H_z was increased in 5 mT step. The blue to red color scale represents lower and higher magnetic field, respectively; when high magnetic contrast appears, the color scale is intentionally saturated at the edge for clarity. Selected frames from the movie are shown Fig. 3a-d of the main text.

Supplementary Movie 4:

Movie of magnetic domains evolution imaged with the SOT between saturated states of 25 nm $CrGeTe_3$ thin film. $\mu_0 H_z$ was ramped up from 0 to 120 mT, then ramped down to -120 mT and back to zero. Magnetic features of ~ 100 nm were observed. By increasing H_z, the domains that are anti-aligned with the field shrink while the ones that are parallel growing. From $\mu_0 H_z \sim 80$ mT, the magnetic contrast drops below 1 mT and the $B_z(x,y)$ stops evolving on that scale. By decreasing the field, the sample's magnetic images remain featureless down to $\mu_0 H_z = 30$ mT, where magnetic domains appear at once. The same behavior was observed for the negative sweep. The frame size was 2×2 μm^2; pixel 30 nm, acquisition time was 5 min/image and H_z was increased in 5 mT step. The blue to red color scale represents lower and higher magnetic field, respectively; when high magnetic contrast appears, the color scale is intentionally saturated at the edge for clarity. Selected frames from the movie are shown Fig. 3e-h of the main text.

Supplementary Movie 5:

Movie of magnetic domains evolution imaged with the SOT between saturated states through an intermediate domain phase in 6 nm $CrGeTe_3$ thin film. $\mu_0 H_z$ was ramped up from 0 to 120 mT, then ramped down to -120 mT and back to zero. At zero field no magnetic features were observed. At $\mu_0 H_z = 20$ mT magnetic domains appear. By increasing the field domains that are anti-aligned with the field shrink while the ones that are parallel growing. From $\mu_0 H_z \sim 45$ mT, the magnetic contrast drops below 1 mT and the $B_z(x,y)$ stops evolving on that scale. By decreasing the field to zero no magnetic features were observed resolving ferromagnetic behavior. The same behavior was observed for the negative sweep. The frame size was 1×1 μm^2; pixel 30 nm, acquisition time was 5 min/image and H_z was increased in 5 mT step. The blue to red color scale represents lower and higher magnetic field, respectively; when high magnetic contrast appears, the color scale is intentionally saturated at the edge for clarity. Selected frames from the movie are shown Fig. 3i-l of the main text.
